# Could simplified stimuli change how the brain performs visual search tasks? A deep neural network study

**DOI:** 10.1167/jov.22.7.3

**Published:** 2022-06-08

**Authors:** David A. Nicholson, Astrid A. Prinz

**Affiliations:** 1 Emory University, Department of Biology, O. Wayne Rollins Research Center, Atlanta, Georgia; 2 Emory University, Department of Biology, O. Wayne Rollins Research Center, Atlanta, Georgia

**Keywords:** visual search, selective visual attention, object recognition, neural networks, deep learning

## Abstract

Visual search is a complex behavior influenced by many factors. To control for these factors, many studies use highly simplified stimuli. However, the statistics of these stimuli are very different from the statistics of the natural images that the human visual system is optimized by evolution and experience to perceive. Could this difference change search behavior? If so, simplified stimuli may contribute to effects typically attributed to cognitive processes, such as selective attention. Here we use deep neural networks to test how optimizing models for the statistics of one distribution of images constrains performance on a task using images from a different distribution. We train four deep neural network architectures on one of three source datasets—natural images, faces, and x-ray images—and then adapt them to a visual search task using simplified stimuli. This adaptation produces models that exhibit performance limitations similar to humans, whereas models trained on the search task alone exhibit no such limitations. However, we also find that deep neural networks trained to classify natural images exhibit similar limitations when adapted to a search task that uses a different set of natural images. Therefore, the distribution of data alone cannot explain this effect. We discuss how future work might integrate an optimization-based approach into existing models of visual search behavior.

## Introduction

Visual search is a complex real-world behavior that we engage in constantly throughout our day. To understand the many factors that influence this behavior (Wolfe & Horowitz, 2017), scientists carry out controlled laboratory experiments. Laboratory visual search tasks are also commonly used to investigate cognitive processes such as attention (Eckstein, 2011; Geisler & Cormack, 2011; Lindsay, 2020; Peelen & Kastner, 2014; Wolfe & Horowitz, 2017) and reward (Anderson, 2016; Maunsell, 2004). A key element of these controlled laboratory experiments is the use of highly simplified stimuli (Wolfe, 1998). These simplified stimuli are designed to experimentally manipulate one factor proposed to influence search behavior while controlling for other factors. This approach has a clear strength. It allows researchers to derive formal mathematical models that are tightly linked to these simplified stimuli, and then arbitrate between models based on the predictions each model makes (Eckstein, 1998; Palmer, 1994; Palmer et al., 2000; Palmer et al., 2011; Wolfe et al., 2010). One possible drawback of this approach is that the features of the simplified, controlled stimuli have very different statistics than the natural images that the human visual system has been optimized by evolution and experience to perceive. Recently in neuroscience there has been renewed concern and discussion about whether simplified behavioral experiments and stimuli may actually limit our ability to understand brain function (Juavinett et al., 2018; Krakauer et al., 2017). Within studies of visual search, the difference between laboratory stimuli and real-world scenes has been recognized, leading to the development of models for “real-world attention” (Peelen & Kastner, 2014). There is even work asking how to design optimal feature detectors given the statistics of natural stimuli (Geisler et al., 2009). That said, we are not aware of any previous work that tests the idea that the difference between simplified search stimuli and natural images may actually give rise to some of the behavior observed during controlled laboratory search experiments. Here we test this idea with deep neural networks (DNNs). DNN models are optimized with large datasets of natural images to perform perceptual tasks such as image classification, and have recently become state-of-the-art models for understanding cognitive functions like object recognition. Below, we further motivate this approach using DNNs, but first we briefly review studies of visual search behavior.

**Figure 1.**
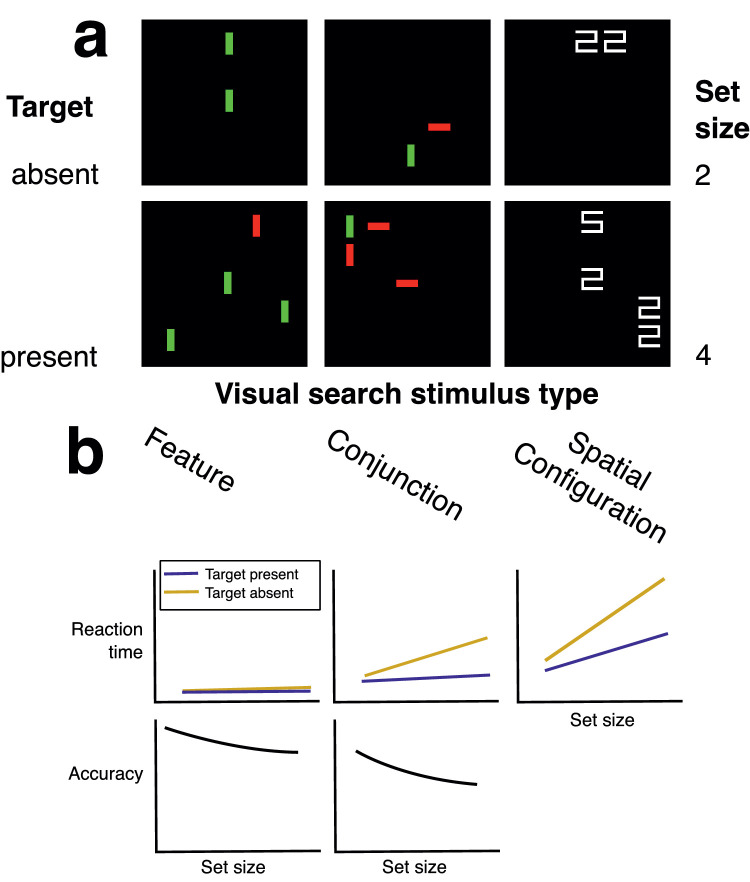
Set size effects are a hallmark finding from laboratory visual search tasks. (a) An example of the simplified displays commonly used in visual search tasks. In the top row of (a), the target is absent and in the bottom row it is present. Displays in each row also have different set sizes (total number of items including target and distractors): on the top row of (a), the set size is two and in the bottom row it is four. (b) Schematic depiction of set size effects, redrawn from (Wolfe et al., 2010) and (Eckstein, 1998)). Effect size varies based on the features that distinguish targets from distractors (shown in columns). In the left column of (a), the target can be distinguished from distractors by a single feature, namely, color; in the middle column, by a conjunction of features, namely, color and orientation (the target is a vertical red bar); in the right column, by a spatial configuration of multiple features.

To address the question of whether the statistics of stimuli used in visual search tasks may change behavior, we consider two experimental paradigms. The first paradigm was designed to study the mechanisms of visual selective attention (Eckstein, 1998; Eckstein et al., 2000; Eckstein, 2011; Palmer et al., 2000; Treisman & Gelade, 1980; Wolfe, 1994; Wolfe et al., 1989; Wolfe & Gray, 2007), using highly simplified stimuli: typically a two-dimensional array of items like those shown in Figure 1. Stimuli like these were originally developed to test feature integration theory (Treisman & Gelade, 1980). One reason for the appeal of this theory was that it was tractable to test with these simplified stimuli (Nakayama & Martini, 2011), using a standardized paradigm (Wolfe, 1998) that has formed the basis of hundreds if not thousands of studies. Participants search the array of items for a target that is distinguished from distractors by one or two parametrically defined features, such as hue, luminance, or orientation. On each trial, the participant reports whether a target is present (Figure 1a, bottom row) or absent (Figure 1a, top row) among the distractors, and the reaction time is measured. The reaction time is then plotted as a function of set size, the total number of items: distractors plus target when present. When reaction time increases as the number of distractors increases, i.e., as a function of set size, this is called a set size effect (Figure 1b, top row). Some studies show each display only briefly, to control for other factors such as eye movement, and these studies may use accuracy as the behavioral measure instead of reaction time (Figure 1b, bottom row). More generally, then, the term set size effect describes any change in a behavioral measure of target detection that depends on increasing the set size. Schematic depictions of results that would indicate set size effects are shown in Figure 1b. Typically, a function is fit to the data, and the fit parameters are used to determine whether a given feature does or does not produce a set size effect. For example, as can be seen in the schematized results in in Figure 1b, the slope is steeper for stimuli where the target is distinguished from distractors by a conjunction of features (middle column) compared to the slope for stimuli where the target is distinguished from distractors by a single feature. These set size effects are taken as evidence for different types of computations thought to be involved in selective attention (Eckstein, 2011; Poder, 2017; Wolfe & Horowitz, 2017).

The second experimental paradigm we consider uses images that are referred to as scenes (Henderson et al., 2009; Neider & Zelinsky, 2008; Rosenholtz, 1999; Torralba, 2005; Wolfe et al., 2011). While the first paradigm was designed specifically to ask how visual search behavior depends on low-level features specified by the experimenter, such as color or orientation, the second is meant to interrogate how search takes place in real-world images. A body of work in this area directly address questions raised by the selective attention literature: how can the concepts of items (Hulleman & Olivers, 2017) and set size (Neider & Zelinsky, 2008) be applied to scenes? Attempts to operationally define the concept of set size for a scene have found that the set size effects are much smaller, indicating that search of real-world scenes is much more efficient (Wolfe et al., 2011). This finding is surprising, given that models of selective attention predict that the visual system would need to process many more low-level features in cluttered scenes (Peelen & Kastner, 2014). Various mechanisms have been proposed to explain these differences in search behavior across tasks (Katti et al., 2017; Neider & Zelinsky, 2008; Peelen & Kastner, 2014; Wolfe et al., 2011). There is general agreement that search of scenes is made more efficient by contextual information not present in simplified search displays (Eckstein, 2017; Wolfe & Horowitz, 2017).

Here we test whether the differences in visual search behavior across these two paradigms might be explained in part by a mismatch between the statistics of simplified stimuli used in some tasks and the statistics of natural images that the visual system is optimized to process. To test this idea, we turn to DNN models. Due to recent successes in engineering DNNs, many researchers in cognition and neuroscience are again making use of these models (Marblestone et al., 2016; Richards et al., 2019; Saxe et al., 2020). DNNs are optimized to perform machine-learning tasks using large datasets, similar to how the visual system is optimized by evolution and development. DNNs are uniquely suited to address the questions we ask here about how the statistics of stimuli interact with the tasks for which the visual system is optimized. There are two strengths of our approach. The first is that we can ask how a model being optimized for one task might constrain how it performs other tasks. As others have argued (Kell & McDermott, 2019), this approach can be seen as analogous to ideal observer models, which have been applied successfully to visual search (Geisler, 2003; Geisler & Cormack, 2011; Kell & McDermott, 2019). Ideal observer models provide insights by adopting a normative approach: proposing a closed-form optimal solution for tasks, and then asking how real-world behavior deviates from the behavior dictated by the optimal solution. Obviously, DNN models do not provide a closed-formed optimal solution for tasks, but the optimization perspective has yielded a significant body of empirical evidence that DNNs perform “near ideally” (Firestone, 2020; Kell & McDermott, 2019), at least as measured with a test dataset that models do not see during training. A second strength of DNNs as models is that they are image computable (Geisler & Cormack, 2011; Yamins et al., 2014), meaning that they accept any image as input. This allows us to measure model behavior across stimulus types. It is difficult if not impossible to compare behavior across stimulus types with selective attention models that are specified (Cooper & Guest, 2014) in terms of items (Hulleman & Olivers, 2017) or human-defined features (Palmer et al., 2000).

Although DNN models as a whole are appropriate to address questions about optimization, it is unclear which type of model and machine learning task to use. Previous work modeling visual search tasks with DNNs has made use of models designed for two distinct computer vision tasks: single-label image classification, and object detection. Single-label image classification is a computer vision task where models assign natural images to a single class, for example, “cat” or “car.” This task can be mapped directly to a yes/no paradigm where a participant classifies an image as “target present” or “target absent.” A DNN architecture known as convolutional neural networks has rapidly become the state of the art for this task. Previous studies have used this family of models to study the experimental paradigm associated with selective attention and have reported set size effects (Poder, 2017; Põder, 2020, 2021). Additionally, the use of convolutional neural network is motivated by previous findings that these models predict behavior during object recognition tasks (Schrimpf et al., 2018; Yamins & DiCarlo, 2016), and other authors have suggested a link between object recognition and visual search (Cohen et al., 2017; Nakayama & Martini, 2011). However, previous work on visual search has also used DNN models of object detection targets (not to be confused with the cognitive ability of object recognition) (Eckstein et al., 2017). It makes sense to consider DNNs for object detection as models of visual search; by definition, visual search involves localizing an object, whereas localization is not typically considered when studying object recognition. In contrast with DNN models for image classification that assign a single label to an image, object detection models produce bounding boxes around many objects of interest. Typically, DNN models for object detection tasks include a convolutional neural network “backbone,” with additional engineered components that use the output of the network to produce candidate bounding boxes. Past studies find that DNN models of object detection employ different strategies than humans (Eckstein et al., 2017). Despite this, it is important to understand which model designed for which task—image classification or object detection—can best address the questions we ask about optimization.

Below, we measure the behavior of DNNs performing laboratory search tasks with simplified stimuli and with real-world scenes and ask how that behavior depends on the types of images used to optimize network parameters. To achieve this goal, we use methods from deep learning to adapt pretrained DNNs to new tasks. First, we test how both object detection models and image classification models behave when they are first trained on natural images and then adapted to a visual search task using simplified stimuli. Consistent with previous work, we find that object detection model performance is at ceiling across set sizes, whereas image classification models exhibit set size effects. We then test whether the set size effect exhibit by DNNs for image classification depends on the dataset they are optimized with, before being adapted to the visual search task. This approach produced results consistent with the idea that the mismatch between the statistics of natural images and simplified stimuli may contribute to performance limitations that participants exhibit in laboratory search tasks. We then ask whether the set size effects disappear when adapting DNNs in the same way to perform the same task with a separate dataset of natural images. Surprisingly, we observe similar set size effects, in contrast with previous studies of search of natural scenes. As we discuss, these results suggest that the optimization viewpoint can contribute to models of visual search behavior, but this work will require careful comparison with the predictions of existing models.

## General methods

### Neural network architectures

In experiments with DNNs for single-label image classification, we utilize four neural network architectures that have been used previously in studies of object recognition, to increase the likelihood that our results are general and not an artifact of any specific architecture. All the models we test are convolutional neural networks (Krizhevsky et al., 2012), where the nonlinearity applied after each layer is the rectified linear activation function (Glorot et al., 2011). Two of the models, AlexNet (Krizhevsky et al., 2012) and VGG16 (Simonyan & Zisserman, 2014), represented key advances in image classification by the computer vision community and were later used in some of the first papers that leveraged DNNs as models of object recognition (Cadieu et al., 2014; Khaligh-Razavi & Kriegeskorte, 2014). The paper describing AlexNet (Krizhevsky et al., 2012) was one of the first to successfully apply deep convolutional neural networks to the task of single-label image classification of the ImageNet dataset (Deng et al., 2009). VGG16 improved on the performance of AlexNet by increasing network depth while using much smaller convolutional filters, particularly in the earlier layers (Simonyan & Zisserman, 2014). The other two architectures, CORnet-S and CORnet-Z, are two DNNs developed to achieve good performance under a metric that captures a model's ability to predict brain activity and behavior during object recognition tasks (Kubilius et al., 2018; Schrimpf et al., 2018). The four convolutional blocks of the CORnet models are meant to correspond to the visual hierarchy in the primate ventral pathway: V1, V2, V4, IT. CORnet-Z (“zero”) is the simplest version of the CORnet architecture, akin to AlexNet with only a single fully-connected layer, whereas CORnet-S (“skip”) makes use of skip connections like those in the ResNet architecture (He et al., 2016) to achieve shallow within-area recurrence. All four architectures make use of an adaptive average pooling layer (He et al., 2015) so that they are image size agnostic. In the next section we provide details of how these DNNs were trained and how we adapted them to visual search tasks.

### Transfer learning

All of our experiments make use of transfer learning (Bengio, 2012; Caruana, 1995, 1997; Kornblith et al., 2019; “Transfer Learning,” 2021; Yosinski et al., 2014), where DNNs are first optimized for one task, such as single-label image classification with the ImageNet dataset, and then adapted to a new task with these pretrained weights.

#### Training

For all four DNN architectures we study, we used publicly available weights that had been optimized for single-label image classification on the ImageNet dataset. We only ever used one set of pretrained weights per architecture and dataset, for all transfer learning experiments described. The loss function for optimizing those weights was standard cross-entropy loss. Loss was minimized with a stochastic gradient descent optimizer over minibatches.

The AlexNet and VGG16 models are the implementations from the torchvision library (Marcel & Rodriguez, 2010). Pretrained weights we used for transfer learning experiments were downloaded programmatically through the library. These weights were trained in a fashion similar to the example script included with that library (https://github.com/pytorch/vision/blob/master/references/classification/train.py). The default training parameters in that script are 90 epochs with a batch size of 32, a learning rate of 0.1, using the stochastic gradient descent optimizer, momentum 0.9, and a learning rate scheduler that decreased the learning rate by 0.1 every 30 epochs. The initial learning rate was decreased to 0.01 for AlexNet and VGG16, because they do not have batch normalization that allows for a higher initial learning rate. For the CORnet models, we use both the implementations and the weights available from the publicly available repository: https://github.com/dicarlolab/CORnet. Weights were trained with the script in that repository. The default training parameters in that script are 20 epochs with a batch size of 256, a learning rate of 0.1, using the stochastic gradient descent optimizer, momentum 0.9, and a learning rate scheduler that decreased the learning rate by 0.1 every 10 epochs.

#### Adaptation

To adapt DNNs to visual search tasks, we hold fixed all parameters in the convolutional layers that are optimized for feature extraction, while updating parameters in the fully-connected decoding layers. We replace the final fully-connected layer used for image classification with a new layer that has an appropriate number of units for the visual search task, and then adapt the model to this task by optimizing for performance with a training set. Again, we use cross-entropy loss with a stochastic gradient descent. For the visual search tasks using simplified displays, the final layer has two output units corresponding to “target present” and “target absent”. DNNs were trained to assign one of these two labels to the displays. On validation steps of training, and at test time, we measured accuracy as simply the number of correctly classified displays (target present or absent) divided by the total number. For the search task using natural images, the number of output units corresponds to the number of classes in the dataset (20 in the case of the Pascal Visual Object Classes [VOC] dataset we use). We used the same transfer learning approach for this search task by selecting one of these candidate classes as a “target,” as described in the main text.

For each model and experimental condition, we generated multiple training replicates (eight replicates for experiment 1, four replicates for experiment 2). This practice means that, for each training replicate, we loaded the one set of pretrained weights into a given DNN architecture and then using that one set of weights we repeated the transfer learning procedure for each replicate. Weights in the final fully-connected layer were the only ones that were randomly initialized for transfer learning experiments. In control experiments, where we did not use pretrained weights, we randomly initialized weights in all layers. We performed this random initialization for each training replicate.

#### Validation of the method

Because our core results hinge on optimizing DNNs with natural images and then performing transfer learning, it was also very important to minimize the possibility that our results were trivially explained by issues with how we performed transfer learning. Before explaining how we minimized this possibility, we emphasize that the method we chose is meant to explicitly test whether the features that DNNs learn to extract from natural images contribute to set size effects. Hence, we froze all weights pretrained to extract features for image classification before adapting weights in the decoding layers to our task of classifying visual search displays. Clearly, freezing weights in the feature extraction layers places a limit on our ability to improve models’ performance. However, even models trained with transfer learning performed quite well as measured on the test set, as shown in the results. We also eliminated the possibility that set size effects arose from other factors of our training method in preliminary experiments (Nicholson & Prinz, 2019). In those preliminary experiments, we examined the effect of imbalance in the dataset, the size of the training set, and hyperparameters such as the learning rate. Essentially, we manually searched for the highest learning rate we could use to ensure that optimization converged and then found that we could combine this with early stopping to prevent overfitting. We also increased the dataset to the largest size possible without generating multiple examples of the same image, to ensure performance was not due to limited training data. Finally, we found in those preliminary experiments that balancing the dataset across visual search set sizes, as we did here, produced the best accuracy. In addition, we took several steps to minimize the possibility that the results presented here were an artifact of our training method. Those steps included logging metrics at each step of training, then visually assessing plots of the logged training histories for evidence of overfitting to the training set, or failure of the optimization to converge. In almost all cases, we saw by plotting the loss values that the optimization converged and that loss also decreased when measured on a validation set that DNN models did not see during training. (We note in the results the few cases where models did not converge.) We also saw that the models achieved high accuracy on the validation set during optimization, indicating that what they learned during training generalized to unseen data. We do not show these training histories here because of space considerations, but they are available in the on-line repository of code that accompanies the paper; see Code availability section in General Methods for link.

#### Validation of the models

**Figure 2.**
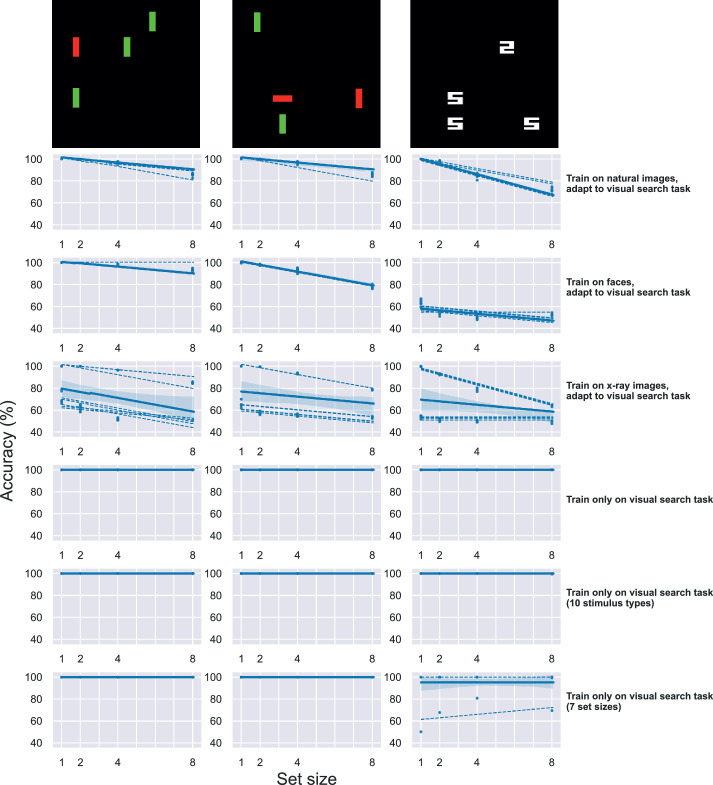
Accuracy as a function of set size, for a single DNN architecture performing a visual search task. Representative results from the VGG16 architecture, performing the task of classifying all search displays as “target present” or “target absent.” Each panel shows accuracy as a function of set size, where accuracy is simply the number of correctly classified displays divided by the total number of displays. Dashed lines indicate mean accuracy across all trials for individual training replicates, and the solid line indicates mean across all trials and replicates. The first three rows show the results for the VGG16 models that were first optimized to classify a separate dataset of images then adapted to this task: first row, natural images (ImageNet), second row, faces (CelebA-Spoof), third row, x-ray images (NIH Chest X-Ray). The second three rows show results for VGG16 models that were trained only on the visual search task: fourth row, same search displays as in the first three rows; fifth row, a dataset with seven more search display types; sixth row, a dataset with four more set sizes. Columns are different stimulus types (example shown at top of column with the target present condition). Stimulus types from left to right are: red vertical line target versus green vertical line distractors; red vertical line target versus red horizontal and green vertical line distractors; white digital two target versus white digital five distractors.

**Figure 3.**
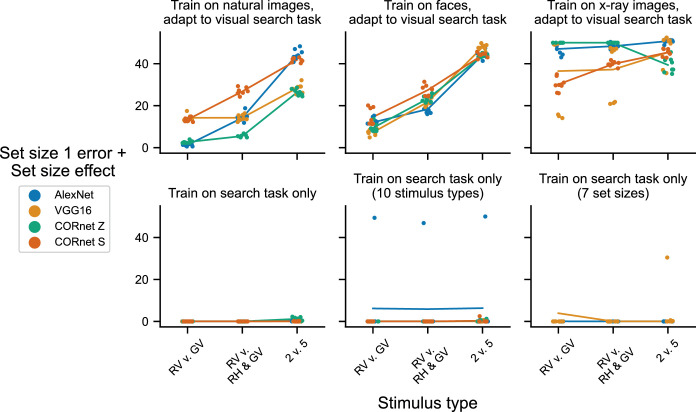
Summary results for four DNN architectures replicating the experiments in Figure 2. Results are summarized as a single value, the error on stimuli with set size 1, plus a “set size effect” computed as the absolute difference between accuracy on the smallest and largest set size. Dots indicate this summary scalar value for one training replicate, and solid lines indicate the mean across all training replicates. Jitter is added on the *x*-axis to make individual dots more visible. Colors correspond to the different DNN architectures: AlexNet (blue), VG16 (orange, same results as those shown in Figure 2), CORnet Z (green) and CORnet S (red). The top row shows results for models that were first trained to classify a separate set of images and then adapted to the visual search task: left column, natural images (ImageNet); middle column, faces (CelebA-Spoof); right column, x-ray images (NIH Chest X-Rays). The bottom row shows results for models that were only ever trained to perform the visual search task: left column, exact same dataset of search displays as used in the top row; middle column, a dataset with seven more search display types; right column, a dataset with four more set sizes.

To assess performance during and after transfer learning, we followed good practices for machine learning (Hastie et al., 2001). These included dividing datasets into training, validation, and test sets. The validation set was used to evaluate the model during training, and the test set was withheld during training and used to measure model behavior afterward. For the search task using simplified displays, all DNN models were trained on a dataset consisting of all different types of stimuli (see, for example, columns in Figure 2), with 1,200 samples for each type. During training, batches were drawn randomly from this dataset, without regard for stimulus type. All results we report used three types, except for experiments that added seven additional stimulus types shown in Figure 3. Stimuli were generated with jitter in the placement of the items, in such a way that guaranteed that there were no repeated images (which might encourage the DNNs to simply memorize the correct answer during training). The maximum number of samples we could generate was 1,200 per stimulus type without repeats, given the parameters we used to create them. For the search task using natural images from the Pascal VOC 2012 dataset (Everingham et al., 2012), we split the data as was done in Ionescu et al. (2016). That is, we used 50% of the Pascal VOC 2012 training-validation set as our training set, 25% as a validation set, and 25% as a test set.

### Code availability

To aid with reproducibility of our experiments, and to make them more accessible to other researchers, we developed a separate software library, visual-search-nets, available at https://github.com/NickleDave/visual-search-nets. We also developed a tool to generate datasets of the simplified visual search stimuli like those we use in Figure 2 and Figure 3, in a format that is convenient for training neural networks, available at https://github.com/NickleDave/searchstims. All configuration files for carrying out experiments, and scripts for generating stimuli, analyzing results, and creating figures, are available in the repository corresponding to this article: https://github.com/NickleDave/Nicholson-Prinz-JOV-DNNs-bio-vis. Libraries, tools, and code for analysis and figures were developed with the following Python libraries: attrs (Schlawack, 2019), numpy (Harris et al., 2020; Walt et al., 2011), scipy (Virtanen et al., 2019), scikit-learn (Grisel et al., 2020; Pedregosa et al., 2011), pandas (team, 2020; McKinney, 2010), matplotlib (Caswell et al., 2020; Hunter, 2007), seaborn (Waskom et al., 2020), jupyter (Kluyver et al., 2016), pingouin (Vallat, 2018), pygame (Schinners, 2019), pytorch (Paszke et al., 2019), statsmodels (Seabold & Perktold, 2010), and torchvision (Marcel & Rodriguez, 2010).

## Results

We test whether optimizing a DNN with one dataset of images constrains its behavior when adapted to a visual search task using a different dataset of images. To adapt DNNs to this task, we employ methods known as transfer learning that are often applied to DNN models (Bengio, 2012; Kornblith et al., 2019; Yosinski et al., 2014) (see Transfer learning in General Methods).

### Comparison of image classification and object detection models

We start by testing which family of DNN models is appropriate to investigate this adaptation phenomenon. To understand which family of DNN model, image classification or object detection, would be appropriate for our simulations, we first assessed the behavior of both when adapted to perform a task with simplified search displays, like those shown in Figure 1. We compare the behavior of a Faster R-CNN model for object detection with a VGG-16 model for image classification. Crucially, the backbone of the Faster R-CNN is the exact same VGG16 model pretrained for image classification on ImageNet (this use of a pretrained backbone is standard for object detection models).

#### Results and discussion

To test the VGG16 model for single-label image classification, we map the yes/no paradigm used in studies of selective attention to a classification task: the model classifies each image with one of two labels: “target present” or “target absent”. As shown in Table 1, when adapted to this task, this VGG16 image classification model did exhibit set size effects (top row).

**Table 1.** Accuracy of VGG16 as an image classification model, and as the backbone an object detection model.

Network	Task	Objectness score threshold	No. of region proposals (pre-NMS)	No. of region proposals (post-NMS)	Overlap threshold	Acc. (set size 1)	Acc. (set size 2)	Acc. (set size 4)	Acc. (set size 8)
VGG16	Image classification	N/A	N/A	N/A	N/A	0.99507813	0.97117188	0.89757813	0.79328125
VGG16	Object detection	0	1000	1000	0.5	1	1	1	1
VGG16	Object detection	0	100	100	0.5	1	1	1	1
VGG16	Object detection	0.95	100	100	0.5	1	0.99875	1	1
VGG16	Object detection	0.95	100	100	0.95	0.9975	0.9975	0.99625	0.985

To test the Faster R-CNN model for object detection, we generated a dataset of the simplified stimuli where each item in a display was annotated with a bounding box. We considered a target item detected when any bounding box overlapped with it by more than 50%, as was done previously, and after initial detection we rejected any further bounding boxes as false positives. When testing the same VGG16 network as the backbone of the Faster R-CNN model, we did not observe set size effects. We found that the model essentially achieves perfect detection of the target item, regardless of the number of distractors, as shown in rows 2 and 3 of Table 1. We only saw a drop in target detection when we reduced the number of candidate bounding boxes by an order of magnitude, raised the objectness score threshold for each bounding box much higher than is typically used, and raised the required overlap for detection to a very stringent 95%. We also repeated the analysis from (Eckstein et al., 2017) and again found with their method of detecting targets that the Faster R-CNN performance was essentially at ceiling for all set sizes (>99%).

These results demonstrate how object detection models are highly engineered to allow for a very high number of initial false positives, so that they can successfully detect all the objects in a scene (Wenkel et al., 2021). It is also consistent with previous work that found that these DNNs employ different strategies than humans performing visual search tasks (Eckstein et al., 2017). Given that these DNNs for object detection are so highly engineered, and have already been shown to exhibit different strategies than humans performing visual search tasks, we did not pursue further studies of these models. In contrast, we found that the exact same VGG-16 model for image classification, pretrained on ImageNet and used as a backbone in the Faster R-CNN, did exhibit a set size-dependent decrease in accuracy when adapted to the yes/no task.

### Constraints on the search task imposed by optimizing with different datasets

The comparison of models led us to proceed with DNN models for image classification. Thus, we take DNNs pretrained for image classification with one of three source datasets, then adapt them to perform the yes/no task, classifying each display as “target present” or “target absent.” We measure the behavior of four DNN architectures used as models of object recognition in the primate ventral visual pathway: AlexNet (Krizhevsky et al., 2012), VGG16 (Simonyan & Zisserman, 2014) used in (Cadieu et al., 2014; Khaligh-Razavi & Kriegeskorte, 2014), and CORnet S and CORnet Z (Schrimpf et al., 2018). We chose these four architectures as a representative sample of DNN-based object recognition models to decrease the likelihood that our results are an artifact of any specific architecture. Essentially, we hold fixed all neural network parameters in the convolutional layers that perform feature extraction. Then we adapt parameters in the fully-connected “decoding” layers on top of the feature extraction layers, replacing the final layer used for image classification with a new layer that has an appropriate number of units for the visual search task. We divide datasets into training, validation, and test subsets, using the validation set to evaluate the model during training, and using the held-out test set to measure model behavior with data not seeing during training.

#### Methods

##### Source datasets

To test whether the effect we observe was specific to models trained on natural images, we trained models on two other source datasets, CelebA-Spoof (Zhang et al., 2020) and the NIH Chest X-Ray dataset (Wang et al., 2017). To train models on the CelebA-Spoof datasets, we modified the script from the torchvision library referenced above to work across datasets. For the CelebA-Spoof dataset, we used the Adam optimizer (see code repository for parameters) and modified the number of classes to two (real image or spoof image). For the NIH X-Ray dataset, we modified another publicly-available code repository to train models for multi-label classification (https://github.com/NickleDave/NIH-Chest-X-Rays-Multi-Label-Image-Classification-In-Pytorch).

#### Results and discussion

We moved on to test more generally whether optimizing the DNN models for image classification with one dataset would constrain performance when adapting them to perform the visual search task. To perform a more general test, we took four neural network architectures and trained each on one of three source datasets, then adapted them to the search task. Representative results for one neural network architecture, VGG16, are shown in Figure 2. As can be seen in the first three rows, when we first optimized VGG16 to classify images—either natural images, faces, or x-ray images—and then adapted the trained model to the visual search task, this strategy resulted in set size effects, where accuracy decreased as the number of distractors increased. It can also be seen that model performance depended in part on the source dataset; predictions of VGG-16 models pretrained on x-ray images were highly variable. We address this issue further below. In contrast with these results, models almost always achieved near-perfect accuracy across set sizes when randomly initialized and then trained only to perform the visual search task, as seen in the bottom three rows. Even when we trained them with an additional seven simplified stimulus types (fifth row) or when we increased the number of different set sizes from four to seven (sixth row), the performance of models trained only on the search task was at the ceiling for most training replicates.

To show that these results as described hold across neural network architectures, we present summary data in Figure 3. In this figure, we reduce model performance to a single number, by taking the error for stimuli with a set size of one and adding to it the absolute difference in accuracy between set size one and set size eight. We combined these two values into a single metric to capture two related phenomena we saw in the results. The first component, error for stimuli with a set size of one, varied across models trained on different datasets, with a clear difference between models trained on x-ray images “compared to models trained on” the other two datasets (top row, Figure 3), as noted above. The second component, the absolute difference in accuracy between set size one and eight, provided a measure of set size effect, similar to the slope typically taken from linear fits to data from experiments with human participants. We did not use the slopes because in one case a linear fit was not appropriate for the data (some replicates of the models trained only on stimuli with seven set sizes produced results that were clearly not linear) (see the right panel in bottom row of Figure 2).

Results in Figure 3 suggest that a DNN for image classification will exhibit behavior with some sort of measurable set size effect when adapted to perform the yes/no task with simplified stimuli after first being trained on any other dataset. For models trained on natural images from the ImageNet dataset or models trained on faces, the set size effect was qualitatively similar to that observed when human subjects perform the task, with a similar ranking of stimulus type to those reported in the literature (Eckstein, 1998; Eckstein et al., 2000; Palmer, 1994). We also observed evidence consistent with the idea that effects might depend in part on the source dataset: models that were first trained on the x-ray images had much higher error even for a set size of 1 (Figure 3, top right panel). Despite this, there was clearly a separable set size effect, regardless of source dataset (Figure 3, top row). Of course, similar set size effects could be obtained trivially with models explicitly designed to discriminate these features. Our goal here was to ask whether the result could be explained in part by optimizing for the statistics of another dataset. To clearly demonstrate this phenomenon, we performed control experiments where models were only ever trained on the yes/no task with simplified stimuli. These models obtained near-perfect accuracy (Figure 3, bottom row), indicating that the main factor contributing to these effects was the adaptation from another dataset. We recognize that, if this were a real behavioral experiment, then it would be poorly designed, because performance would be at the ceiling in almost all conditions. We reiterate that our goal here was simply to show how the behavior of DNN models depends on optimization, and not to make any broader claims about the intrinsic ranking of stimuli and the efficiency with which they can be searched.

### Additional control experiments

We also carried out additional control experiments that we summarize briefly. The results are provided with the code repository associated with this article (see the link in the General Methods) but, are not shown because of space considerations. To rule out the possibility that set size effects are a result of the transfer learning method we used, we trained the same DNNs to first classify the simplified stimuli, where each stimulus type was one class, and then repeated the transfer learning experiment, adapting the pretrained models to the task of classifying all stimulus types as either target present or target absent. Models again achieved near-perfect accuracy, similar to results shown in the bottom row of Figure 3, indicating that transfer learning alone does not produce set size effects. We also carried out transfer learning experiments with AlexNet and VGG16 architectures pretrained on other datasets. We tested models trained on Stylized ImageNet, a dataset that has been used to make DNNs less sensitive to texture, and more responsive to shape, as humans are (Geirhos et al., 2019). These models still exhibited set size effects. In addition, we trained models on the Clipart domain of the DomainNet dataset. These models again exhibited set size effects. A final concern that might be raised about our results is that the simplified stimuli might change the statistics of activations within the hidden layers of the neural networks in a way that impedes networks’ ability to learn the task. For example, the black backgrounds might produce lower activations on average than the activations produced by full-color images from ImageNet used when training models for object recognition. To address this concern, we carried out a control experiment where we produced the same set of simplified stimulus types, only with a white background instead of black, and we repeated the training with the AlexNet model. We again saw that AlexNet models pretrained on ImageNet exhibited set size effects, whereas AlexNet models trained from randomly initialized weights were able to achieve very high accuracy on the same task.

### Psychophysics experiment

We sought to understand what gave rise to the difference in performance we saw when optimizing DNNs only with simplified search stimuli versus optimizing them with real-world, natural images. Based on our results, we predicted that the types of images used to optimize DNNs would impact the models’ ability to generalize. To test this, we adapted a psychophysics-based approach that has been used previously when studying selective attention. Researchers taking this approach have shown that they can control for target–distractor similarity and still detect set size effects (Palmer, 1994; Palmer et al., 1993, 2000). Experimentally, they vary the target–distractor similarity across sessions, and then fit a psychometric curve for each display set size, where performance is a function of discriminability. Finally, they find some fixed threshold value, e.g., the discriminability that yielded 75% accuracy, and plot those thresholds as a function of the set size. In this way, the analysis tests whether set size effects persist even when behavior is measured at a fixed discriminability threshold.

#### Methods

To further interrogate model behavior, we perform psychophysics experiments with DNNs adapted to the visual search task that uses simplified search displays. In order to do so, we generated additional datasets of search displays where target-distractor discriminability varied. We tested DNNs trained on 10 stimulus types with two of those stimulus types: (1) red vertical rectangle target versus green vertical rectangle distractors, and (2) T rotated 90° target versus T (not rotated) distractors. For the first stimulus type, we varied the color of the target from green (0% discriminable) to red (100% discriminable). For the second type, we varied the rotation of the target, from 0° (i.e., not rotated, 0% discriminable) to 90°. We chose 12 points between 0% and 100% discriminability for both targets, and at each point generated 256 unique stimulus displays for all four set sizes, a total of 1,024 for each discriminability level. Then we used these datasets to measure accuracy at each level of discriminability.

We then fit a psychometric function to the results:

$$\begin{array}{c} P\left(x\right)=\gamma +\left(1-\gamma \right){\left(1+e^{-\left(x-\alpha \right)/\beta }\right)}^{-1}. \end{array}$$

We chose this function simply because it is widely used (Strasburger, 2001; Wichmann & Hill, 2001). It is not meant to imply anything about how DNNs process stimuli (e.g., as the Weibull function was used to model a nonlinear transducer in (May & Solomon, 2013)). The function we use can be seen as a logistic that provides a sigmoid shape, combined with the term (*x*–α)/β used to standardize normally distributed data (Hill, 2005). For the specific function we use, α is the 75% threshold and β is a scaling factor inversely related to the slope (Hill, 2005; Strasburger, 2001). When reporting results we refer to β as the “slope” as is common convention (Strasburger, 2001), although both α and β affect the slope when the fit is performed on a linear abscissa (May & Solomon, 2013) (not log transformed), as we do here. To estimate parameters α and β we fit results from the psychophysics experiments, using the scipy.optimize.curve_fit function, with initial values (α = 0.5, β = 0.05) and the guessing rate γ set to performance at chance, 0.5, in all cases.

After performing the fitting, we ran a regression on the log(threshold)–log(set size) values, as was done in Palmer (1994) and Palmer et al. (2000). Following their methods, we used the fit psychometric function to determine the discriminability at which DNNs correctly classified 75% of search displays, and used that value of discriminability as the threshold when we ran the regression.

#### Results and discussion

**Figure 4.**
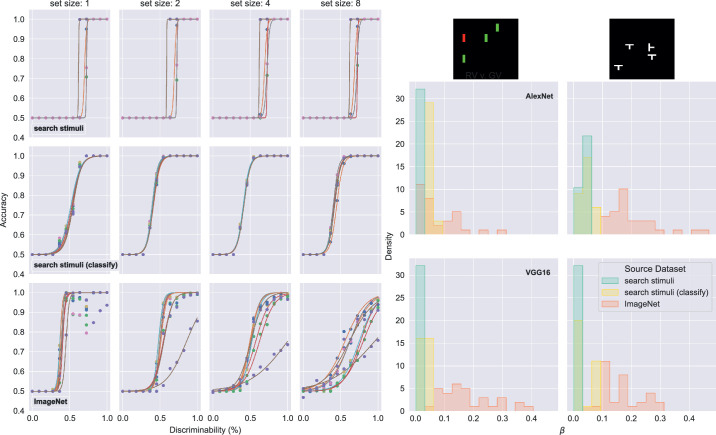
Results of psychophysics experiments. (Left) Representative examples of fits of psychometric curves. All curves are from AlexNet models with accuracy (*y* axis) measured on a dataset of search displays where the target is distinguished from the distractor by color, and the discriminability was varied from 0 (both target and distractor are green) to 100 (target is red, as in the original training set). Circular markers indicate measured accuracy. Each line is a fit to those accuracies from one training replicate. (Right) A histogram of beta parameters from psychometric function fits. Fill colors in bars indicate source dataset DNNs were trained on, if any, before being adapted to the task using search display stimuli: “ImageNet” models were first optimized to classify natural images, “search stimuli (classify)” were first optimized to classify the types of simplified search displays, and “search stimuli” were only ever trained to classify all such displays as either “target present” or “absent.”

We hypothesized that the type of images used to optimize DNNs would impact their sensitivity to targets, as measured by the parameters of the fit psychometric functions. To carry out psychophysics experiments with the trained DNNs, we generated additional datasets of search displays where target-distractor discriminability varied. For each training replicate, we measured accuracy at each level of discriminability and then fit a standard psychometric function to the results. In the first panel of Figure 4, we show the results of performing these fits with AlexNet models optimized with different types of images. After performing the fitting, we ran a regression on the log(threshold)*-*log(set size) values as was done in Palmer (1994) and Palmer et al. (2000). When replicating their analysis, we did not see any consistent difference in set size effects that depended on image types used during optimization (results not shown). However, we did observe a clear difference in the fit parameters. For models trained on just the simplified stimuli, we saw qualitatively that the fits essentially produced step functions (Figure 4, left panel, top and middle row), whereas the fits for models trained on any real images were more like those expected for a well-calibrated psychophysics experiment (Figure 4, left panel, bottom row). This qualitative difference was matched quantitatively by a clear difference in the values of the fit β parameter, which contributes to the slope and shape of the fit psychometric function (May & Solomon, 2013; Strasburger, 2001). As shown in the second panel of Figure 4, models trained with just simplified stimuli had β parameter values of less than 0.1, regardless of set size, whereas the models that were first optimized to classify ImageNet images had a range of β parameter values up to 0.4 that varied with the set size.

We observed a clear difference between models trained only on the simplified stimuli compared with models trained on natural images. This difference could be seen in the curves and was clear from the β parameters produced by fits. The difference in β parameter values demonstrates that DNNs trained on simplified stimuli alone are highly tuned to very specific features. This outcome is not simply overfitting in its standard sense: despite this tuning, DNNs trained on simplified stimuli achieve near perfect accuracy on a large test set not seen during the training time (Figure 3). In a world of simplified search displays, these models would generalize perfectly. By comparison, DNNs optimized with the natural images in the ImageNet dataset seem to be tuned more broadly.

We also carried out other analyses that like this psychometric experiment were meant to identify a mechanism that might explain the differences in behavior we observed between models trained on natural images and models trained on only the simplified search displays. These analyses included measurements of learned kernel similarity, distance between hidden layer activations, and Rényi entropy (Wickstrøm et al., 2019). None of them provided a measure that consistently explained differences across neural network architectures and training sets. We include the analyses with the on-line code repository associated with the article, but omit the results here. Based on these results, we suggest that approaches from psychophysics applied to DNNs may prove more informative than explainable AI–type approaches, a point we return to in the discussion.

### Visual search task with natural images

**Figure 5.**
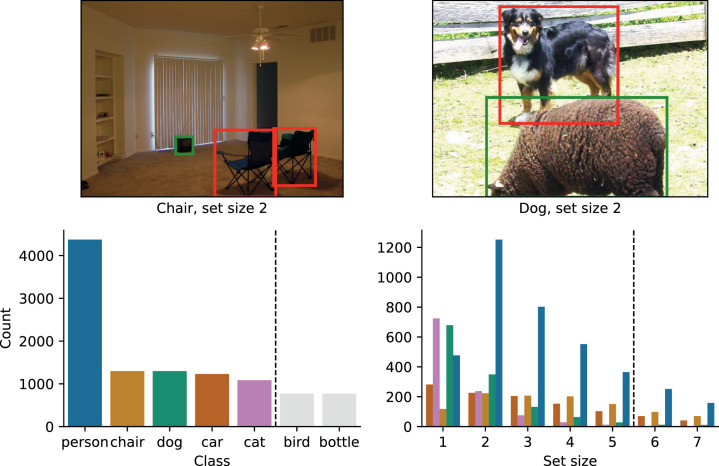
Modifying the Pascal VOC dataset to perform the same visual search task as in experiment 1. The top row shows example images from VOC along with bounding box annotations (red and green boxes). (Left) An example that we used as a chair class. Bounding box annotations are shown here for reference; for experiments we assigned this image a single label, “target present,” as we did in experiment 1. (Right) An example of “target present” for the dog class, and the green boxes correspond with distractor classes. The bottom row presents summary statistics of the dataset. As shown in the left of the bottom row, we ranked the 20 classes by their occurrence and chose the five most frequently occurring (left side of the vertical dashed line). This gave us five candidate “targets” with different distributions of “set sizes” in the annotation, where set size is computed as the total number of annotated bounding boxes in each image. The resulting distributions of set sizes are shown in the right panel of the bottom row. Because there were some candidate targets for which there were no examples with a set size of greater than five (right side of the vertical dashed line) we only considered set sizes of one to five when analyzing results.

We again ask how optimizing for one dataset constrains performance on another dataset, but with a crucial difference: here in both cases the datasets are of natural images. Logically, it makes sense to ask whether we observe similar effects when applying the exact same approach, but with two datasets that are arguably drawn from the same underlying distribution. Additionally, we design the task so that we can directly compare our results with previous work on visual search in scenes, which used an operational definition of set size for the scenes to test for set size effects (Neider & Zelinsky, 2008; Wolfe et al., 2011). This previous work found that searches of natural scenes were much more efficient than search of simplified two-dimensional arrays of items. Here we obtain a measure of set size for natural images by making use of a benchmark dataset that is designed for object detection, the Pascal VOC dataset (Everingham et al., 2012), that has also been used in a previous study of visual search (Ionescu et al., 2016). We compute a set size for images in this dataset by simply counting the number of annotated bounding boxes in each image (see examples in Figure 5). As noted in previous work, such a measure is an imperfect heuristic (Wolfe et al., 2011), but here it allows us to directly compare the behavior of a single model across the two experimental paradigms.

#### Methods

We sought to use the exact same visual search task used in experiment 1 so that we could better test whether the effects we saw can be attributed to the difference in statistics between datasets. To achieve this, we labeled all images in the Pascal VOC dataset as “target present” or “target absent”, just as we did with the simplified search displays in experiment 1. We chose the five most frequently occurring classes in Pascal VOC as candidate targets: person, chair, dog, cat, and car (see bottom left panel of Figure 5). This strategy produced five datasets that all shared the same images but had different “target present” or “target absent” labels each image depending on the target class, as derived from the original Pascal VOC annotation. We also assigned each image a set size by counting the number of annotated bounding boxes. The set sizes were constant across the five candidate target classes, but the number of “target present” images for each set size varied by candidate class. Using this definition of set size, we could only go up to a set size of five and still have some “target present” images for each candidate target class, as shown in the bottom right panel of Figure 5. Thus, we limit our analysis to set sizes of one to five. Note also that the varying number of images per set size across candidate target classes shows that the datasets were not carefully balanced across set sizes (if they were balanced, this might be expected to minimize the possibility that the networks showed set size effects). Given these five datasets, we then adapted DNN models trained to classify ImageNet images to perform this task, using the exact same model weights as in experiment 1.

#### Results and discussion

**Figure 6.**
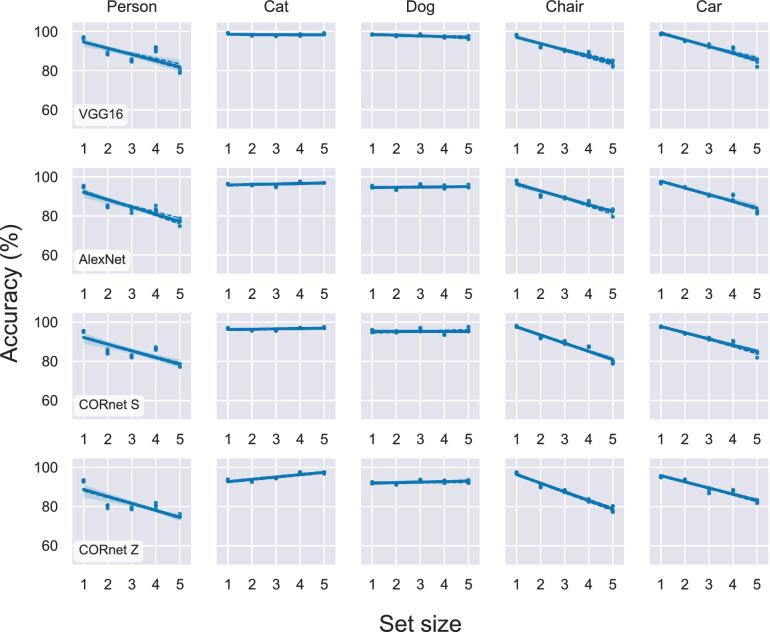
Accuracy as a function of set size for yes/no visual search task using real-world images. As in experiment 1, the task was to classify each image as “target present” or “target absent,” but in this case the target was a real-world class, one of “person,” “chair,” “dog,” “car,” or “horse,” chosen as described in Experiment 2 methods and shown in Figure 5. Models pretrained to classify ImageNet were adapted to this task using the Pascal VOC dataset, with the “target present” or “target absent” labels changed for each simulation, according to which class was designated the target. Each panel shows mean accuracy across four training replicates. Standard deviation was relatively small across replicates (not visible in plot). Each column shows results for one candidate target, ranked in order from most to least frequent as in Figure 5. Each row presents results for one neural network architecture.

As before, we tested how adapting the same four neural network architectures to a new dataset constrained their performance. We found that DNNs did exhibit set size effects when adapting to a different dataset of natural images, as shown in Figure 6. There was a clear decrease in accuracy between set sizes of one and five for three of the candidate target classes: person, chair, and car. However, this outcome was not true for all candidate targets. Qualitatively, there appeared to be no decrease in accuracy for the dog class, and surprisingly for the cat class, the accuracy improved slightly for larger set sizes. This behavior was consistent across all four neural network architectures, as can be seen across rows in Figure 6.

It is surprising that we did observe set size effects when transferring models from one set of natural images to another. Based on previous research on transfer learning, it is to be expected that models perform well when adapted to another dataset that is in the same domain (Kornblith et al., 2019; Yosinski et al., 2014). One alternate explanation for this result could be the relatively limited amount of data in the Pascal VOC dataset. We used the “train-val” split of the 2012 version of the dataset (as was done in Ionescu et al. [2016]), which gave us approximately 6,000 images for training. By comparison, the datasets we generated of two-dimensional search arrays contained at least 38,400 images (and more in cases where we increased the number of stimulus types or set sizes). Unfortunately, ruling out this alternate explanation would require a much larger dataset of natural images that are hand-annotated with bounding boxes.

### Statistical comparisons between datasets

**Figure 7.**
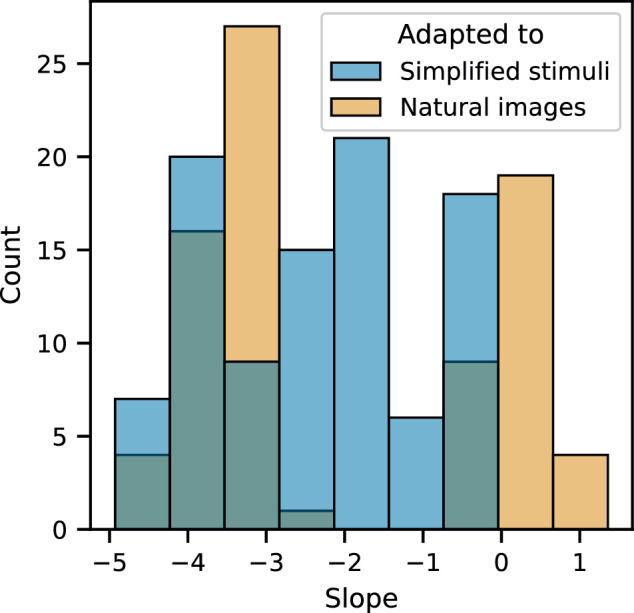
Distribution of slopes fit to data from the two visual search tasks. Slopes are from least squares linear regressions fit directly to accuracy as a function of set size. Hue indicates the dataset used in the visual search tasks.

Lastly, we tested whether we could detect a statistical difference in the effects we saw between tasks, and whether this difference depended on the dataset that the models were adapted to. To test for this, we considered only models that were first trained on natural images (the ImageNet dataset), and asked whether there was a difference in set size effect that depended on the dataset used in the visual search task, i.e., the simplified search displays or natural images from the Pascal VOC dataset. We performed simple linear regressions on accuracy as a function of set size, and compared the slopes from models adapted to the simplified search displays with slopes from models adapted to the natural images. The distribution of slopes is shown in Figure 7. To test for a difference, we performed a nonparametric one-sided Mann–Whitney *U* test. The alternative hypothesis was that slopes for the models adapted to the task with simplified stimuli were less than slopes for models adapted to the task. (Note that most slopes were negative because accuracy decreased as a function of set size, as shown in Figure 7.) The test was not significant (*p* = 0.14).

## General discussion

We asked whether stimulus types used in visual search tasks may influence performance. More specifically, we asked whether the statistics of simplified displays used to test theories of selective attention may be mismatched with the statistics that the visual system is optimized to perceive, by evolution and by experience with natural images. To test this idea, we made use of deep neural networks (DNNs). Because DNNs are optimized for task performance in a data-driven way, they make it possible to test how optimizing for one task, using one type of stimulus, may impose constraints on other tasks that use different types of stimuli. In addition, DNNs are image computable, meaning that we might be able to use them to account for behavior across all types of images used in visual search tasks. First, we tested which families of DNN model were appropriate to test our hypothesis. We found that DNNs for object detection were at ceiling for all set sizes (Table 1), because they are carefully engineered to detect all objects in a scene. This finding is consistent with previous work that found that DNNs for objecy detection employ different strategies than humans. In contrast, a DNN for single-label image classification did exhibit human-like performance limitations when adapted to the visual search task using simplified stimuli, as has been shown in other studies. To test the generality of this finding, we tested three other DNN architectures for single-label image classification. We first trained each architecture on one of three source datasets—natural images from ImageNet, faces from the CelebA-Spoof dataset, or x-ray images from the NIH Chest X-Ray dataset—and then adapted them to perform the visual search task that uses simplified displays We found that the behavior of these DNNs qualitatively resembled that of human participants performing the task, resulting in measurable set size effects (Figure 2) that are typically attributed to selective attention mechanisms. To further test whether set size effects resulted from optimizing DNNs with natural images, we carried out separate experiments where we trained the same DNN architectures on simplified displays alone, instead of using weights pretrained for image classification. When trained this way, the exact same DNN architectures are capable of performing the task with near-perfect accuracy (Figure 3). Lastly, we tested whether this effect disappeared when we adapted DNNs in the same way to a visual search task where the dataset was again natural images (Figure 5). In this case, we did still see set size effects (Figure 6), and, when we compared slopes fit to the data from the two different tasks (Figure 7), we were unable to find a significant difference between the simplified stimuli and the natural images. Taken together, our results provide some evidence that a mismatch between statistics of stimuli used in search tasks could contribute to visual search behavior. However, they also suggest that other factors in our modeling approach could produce similar effects, such as limited data when adapting models to the visual search task. A better understanding of these inconsistencies will need to be resolved by more fine-grained comparisons of DNN model behavior with human behavior. Such comparisons are both necessary and informative (Firestone, 2020; Funke et al., 2020; Geirhos et al., 2020; Kim et al., 2020).

This need to further test empirically points to potential weaknesses of our findings. One weakness of our approach here is that the DNN models we used cannot account for other behavioral measures, the most crucial of which is reaction time. We discuss how to extend DNN models to account for reaction times below. Here we point out that, even though we cannot account for reaction times directly, our results at least suggest that DNN models have the potential to account for behavior across visual search tasks. While we cannot make strong claims about our findings across tasks, these results do suggest DNNs can address a weakness of selective models of attention, which usually neglect the problem of feature extraction, and so are not easily extended to account for behavior across multiple stimulus types. It should be said there are models of visual search that do propose explicit feature extraction mechanisms (Akbas & Eckstein, 2017; Zelinsky, 2008), including the modeling of localization and spatial uncertainty (Burgess & Ghandeharian, 1984; Swensson & Judy, 1981), which is neglected by commonly used DNN models. Future work modeling visual search with DNN architectures should draw from that literature.

Another weakness of our findings relates to the set size effects we observed when analyzing DNN behavior. Set size effects alone do not provide sufficient support for any mechanism that claims to account for performance limitations (Kristjánsson, 2015; Nakayama & Martini, 2011). For this reason, researchers have turned to multiple measures, such as comparisons between distributions (Wolfe et al., 2010), to arbitrate between the proposed mechanisms. It is very possible that measuring multiple aspects of the behavior of the DNN models we tested here may reveal differences in how they solve visual search tasks compared to humans. The results we obtained by performing psychometric experiments on trained DNNs (Figure 4) hint at this. A similar approach may prove useful in future studies.

A first step toward addressing some of the potential weaknesses of DNN models we have just outlined would be to extend these models so that they also produce reaction times. This practice would enable researchers to test whether a single model accounts for results not just across stimuli, but also across the different protocols for performing visual search tasks. There are several methods for extending DNN models so they produce reaction times. The first would be to use recurrent neural networks, which carry out a computation for a specified number of time steps *t*, as has been done for studies of object recognition (Kar et al., 2019; Kietzmann et al., 2019; Nayebi et al., 2018; Spoerer et al., 2017). In general, these studies find that recurrence conveys an advantage in terms of predicting neural activity and behavior. Another solution would be to add computations to DNNs from modeling studies of visual search, computations that also produce reaction times, such as a winner-take-all or diffusion-drift mechanisms (Moran et al., 2013; Narbutas et al., 2017). Although these mechanisms could be applied to DNN models, the models would always produce the same reaction time given a particular image, because DNN output is deterministic (at least, at inference time, ignoring things like stochastic dropout often used during training). In contrast, human subjects produce a distribution of reaction times across trials (Wolfe et al., 2010). Addressing all of these factors may require adopting a different theoretical framework. For example, building models within the Neural Engineering Framework (Eliasmith & Anderson, 2003; Eliasmith & Stewart, 2011) would make it possible to augment DNNs tested here (Rasmussen, 2019) with winner-take-all mechanisms (Gosmann et al., 2017) and variable neural activity (Bekolay et al., 2014; Hunsberger et al., 2014; Hunsberger, 2018), both of which are thought to be important for visual search behavior but are missing from standard DNN models.

As stated in the Introduction, our experiments were mainly concerned with whether the stimulus type used in visual search tasks might change behavior in a way that is attributed to other factors, such as selective attention mechanisms. Others have argued that one way to reconcile results across experimental paradigms would be to explicitly incorporate probabilistic computations into models of visual search behavior (Eckstein, 2017). Our results are wholly consistent with the claim that visual search behavior across experimental paradigms can be accounted for by probabilistic models, without invoking causal cognitive processes like attention (Anderson, 2011; Hommel et al., 2019; Vincent, 2015). Within such a modeling framework, the set size effects we observed would be explained by the priors learned from the datasets used to optimize models before adapting them to visual search tasks. While we acknowledge this, we insist that it is important to understand how the visual system being optimized for one aspect of behavior imposes constraints on other aspects (Kell & McDermott, 2019), and we suggest that data-driven optimization provides a tractable method to address this question. Our results represent a first glimpse of such an approach. We presented evidence consistent with the idea that the visual system being optimized for the statistics of natural images might impose constraints when faced with a task that uses stimuli drawn from a different distribution. It is also clear from our results that there are very real differences between the way DNNs are optimized for machine learning tasks and the way the visual system is optimized by evolution and experience. Future work will need to better align data-driven optimization of models with what is known about development of the visual system (Smith & Slone, 2017). More broadly, the study of visual search behavior may benefit from direct comparison of predictions made by models we tested here with existing models. The ready availability of user-friendly software for building DNNs has increased usage of these models. In contrast, there are many descriptions of well-known conceptual models of visual search behavior (Wolfe, 2020; Wolfe, 1994, 2021; Wolfe et al., 1989; Wolfe & Gray, 2007), but very few widely available computational implementations (Moran et al., 2013, 2016) of those same models. A virtuous cycle of implementing these models and comparing their behavior with that of DNN-based models would drive theory forward (Guest & Martin, 2020) and permit a more nuanced understanding of what we mean when we say that visual search behavior is “optimal” (Eckstein, 2017; Geisler, 2011; Geisler et al., 2009; Geisler & Cormack, 2011; Kell & McDermott, 2019; Richards et al., 2019; Vincent, 2015).

## Conclusions

We asked whether stimulus types used in visual search tasks may influence performance, because of how well they match the statistics of the natural images the human visual system is optimized to process. To test this idea, we leveraged the strengths of DNN models that are optimized for task performance with large datasets of images. We demonstrated that DNNs exhibit a hallmark effect seen when participants search simplified stimulus types often used in laboratory tasks, and this effect results from optimizing DNNs with another dataset before adapting them to the visual search task. However, we observed similar behavior when adapting DNNs trained on natural images to a visual search task that used a separate dataset of natural images. Our findings are consistent with the idea that optimization for one task can impose constraints on other tasks, but they also raise questions about how optimization of DNNs is different from development of the visual system, that will need to be addressed by future work.
